# Type 1, 2, and 1/2-Hybrid IncC Plasmids From China

**DOI:** 10.3389/fmicb.2019.02508

**Published:** 2019-11-15

**Authors:** Qiaoxiang Cheng, Xiaoyuan Jiang, Yanan Xu, Lingfei Hu, Wenbo Luo, Zhe Yin, Huixia Gao, Wenhui Yang, Huiying Yang, Yuee Zhao, Xiaodong Zhao, Dongsheng Zhou, Erhei Dai

**Affiliations:** ^1^Department of Clinical Laboratory Medicine, Hebei Medical University, Shijiazhuang, China; ^2^Department of Laboratory Medicine, The Fifth Hospital of Shijiazhuang, Hebei Medical University, Shijiazhuang, China; ^3^State Key Laboratory of Pathogen and Biosecurity, Beijing Institute of Microbiology and Epidemiology, Beijing, China

**Keywords:** plasmids, IncC, multidrug resistance, resistance islands, Tn*6395*, In2-76, IS*Ecl10*

## Abstract

A collection of 11 IncC plasmids from China were fully sequenced herein and compared with reference plasmids pR148 and pR55. These 13 plasmids could be assigned into three different subgroups: type 1, type 2, and type 1/2 hybrid. Type 1/2-hybrid plasmids most likely emerged from homologous recombination between type 1 and type 2 plasmids. Different IncC plasmids had evolved to acquire quite different profiles of accessory modules and thus different collections of resistance genes. The accessory resistance modules included not only the *bla*_CMY_-carrying region, the ARI-A island, and the ARI-B island, but also various additional kinds of resistance islands such as the *bla*_CTX–M_-carrying regions and the MDR regions. Insertion of accessory modules was sometimes accompanied by deletion, inversion, and translocation of surrounding backbone regions. pR148 and pR55 were confirmed to have the most complete backbones for type 1 and type 2, respectively. This was the first report of a *bla*_IMP–__8_-carrying IncC plasmid, and that of three novel mobile elements: a Tn*1696*-derived unit transposon Tn*6395*, a class 2 integron In2-76, and an insertion sequence IS*Ecl10*.

## Introduction

Plasmids of IncA and IncC incompatibility groups have been combined into IncA/C since 1970s, but IncA and IncC groups are essentially compatible (Ambrose et al., 2018) and have significant nucleotide divergence across their backbones (Harmer and Hall, 2015) and, thus, IncA [reference plasmid pRA1 (Fricke et al., 2009)] and IncC should be separated from IncA/C (Harmer et al., 2017). IncC plasmids have two distinct types, namely, type 1 and type 2 (Harmer and Hall, 2014), which are represented by reference plasmids pR148 (Del Castillo et al., 2013), and pR55 (Benoît et al., 2012), respectively. These two types can be distinguished by two genes of substitution (*orf1832* in type 1 or *orf1847* in type 2, and *rhs1* in type 1 or *rhs2* in type 2) and two small intergenic insertions (i1 and i2 found in type 2 rather than type 1) (Harmer and Hall, 2014).

A large array of antibiotic resistance genes have been found in IncC plasmids and are clustered in various accessory modules inserted at various sites of the IncC backbones (Harmer and Hall, 2015). IncC plasmids contain at least three hotspots for integration of the *bla*_CMY_-carrying region and two antibiotic resistance islands designated ARI-A and ARI-B. The *bla*_CMY_-carrying region and ARI-A are frequently found in type 1 plasmids but not type 2; both type 1 and type 2 plasmids carry ARI-B, but this island is not always present in these plasmids (Harmer and Hall, 2015).

Our previous study (Ma et al., 2017) has shown considerable genomic variation in a collection of three type 1 IncC plasmids from China together with pR148. This follow-up study presents the complete nucleotide sequences of 11 new IncC plasmids from China and a further comprehensive genomic comparison of these 11 plasmids together with pR148 and pR55, providing a deeper insight into parallel evolution of IncC plasmids from China.

## Materials and Methods

### Bacterial Strains

The 11 isolates (Supplementary Table S1) were recovered from the sputum or blood specimens of 11 different patients with nosocomial infections in 10 Chinese public hospitals from 2012 to 2016. Bacterial species identification was performed using genome sequence-based average nucleotide identity analysis (Richter and Rossello-Mora, 2009).

### Conjugal Transfer

Plasmid conjugal transfer experiments were carried out with rifampin-resistant *Escherichia coli* EC600 or sodium azide-resistant *E. coli* J53 being used as a recipient and each of the QD1501, A2293, and 205880 isolates as a donor. Three milliliters of overnight cultures of each of donor and recipient bacteria were mixed together, harvested, and resuspended in 80 μl of Brain Heart Infusion (BHI) broth (BD Biosciences). The mixture was spotted on a 1 cm^2^ hydrophilic nylon membrane filter with a 0.45 μm pore size (Millipore) that was placed on a BHI agar (BD Biosciences) plate and then incubated for mating at 37°C for 12 to 18 h. Bacteria were washed from filter membrane and spotted on Muller-Hinton (MH) agar (BD Biosciences) plates, for selecting an *E. coli* transconjugant carrying *bla*_NDM_ (pQD1501-Ct1), *bla*_CTX–M_ (pA2293-Ct2), or *bla*_CMY_ (p205880-Ct1/2). 200 μg/ml sodium azide (for J53) or 1000 μg/ml rifampin (for EC600), together with 4 μg/ml imipenem (for *bla*_NDM_) or 200 μg/ml ampicillin (for *bla*_CTX–M_ or *bla*_CMY_), was for *E. coli* transconjugant selection.

### Sequencing and Sequence Assembly

Genomic DNA was isolated from each of the above 11 isolates using an UltraClean Microbial Kit (Qiagen, NW, Germany). The genomic DNA of strains 427113, T5282, and 397108 was sequenced from mate-pair libraries with an average insert size of 5 kb (ranging from 2 to 10 kb) using a MiSeq sequencer (Illumina, CA, United States) and DNA contigs were assembled based on their contig coverages using *Newbler* 2.6 (Nederbragt, 2014). Quality control, removing adapters, and low quality reads were performed using *Trimmomatic* 0.36 (Bolger et al., 2014). The filtered clean reads were then assembled using *Newbler* 2.6 (Nederbragt, 2014), followed by extraction of the consensus sequence with *CLC Genomics Workbench* 3.0 (Qiagen Bioinformatics). *Gapfiller* V1.11 (Boetzer and Pirovano, 2012) was used for gap closure. For all the other eight isolates, genome sequencing was performed with a sheared DNA library with an average size of 15 kb (ranging from 10 to 20 kb) on a PacBio RSII sequencer (Pacific Biosciences, CA, United States), as well as a paired-end library with an average insert size of 400 bp (ranging from 150 to 600 kb) on a HiSeq sequencer (Illumina, CA, United States). The paired-end short Illumina reads were used to correct the long PacBio reads utilizing *proovread* (Hackl et al., 2014), and then the corrected PacBio reads were assembled *de novo* utilizing *SMARTdenovo*^1^.

### Sequence Annotation and Comparison

Open reading frames (ORFs) and pseudogenes were predicted using *RAST* 2.0 (Brettin et al., 2015) combined with *BLASTP/BLASTN* (Boratyn et al., 2013) searches against the *UniProtKB/Swiss-Prot* database (Boutet et al., 2016) and the *RefSeq* database (O’Leary et al., 2016). Annotation of resistance genes, mobile elements, and other features was carried out using the online databases including *CARD* (Jia et al., 2017), *ResFinder* (Zankari et al., 2012), *ISfinder* (Siguier et al., 2006), *INTEGRALL* (Moura et al., 2009), and *Tn Number Registry* (Roberts et al., 2008). Multiple and pairwise sequence comparisons were performed using *MUSCLE* 3.8.31 (Edgar, 2004) and *BLASTN*, respectively. Gene organization diagrams were drawn in *Inkscape* 0.48.1^2^. Heatmaps were plotted with *MeV* 4.9.0 (Saeed et al., 2003).

### Phylogenetic Analysis

The backbone regions of indicative plasmids were aligned using *MUMmer* 3.0 (Kurtz et al., 2004). Inference of homologous recombination was performed using *ClonalFrameML* (Didelot and Wilson, 2015) to remove recombination-associated single-nucleotide polymorphisms (SNPs). A maximum-likelihood tree was constructed from recombination-free SNPs using *MEGA7* (Kumar et al., 2016) with a bootstrap iteration of 1000.

### Antimicrobial Susceptibility Testing

Bacterial antimicrobial susceptibility was tested by BioMérieux VITEK 2 and interpreted as per the 2017 Clinical and Laboratory Standards Institute (CLSI) guidelines (CLSI, 2017).

### Nucleotide Sequence Accession Numbers

The complete nucleotide sequences of plasmids p24845-Ct2, p205880-Ct1/2, pT5282-Ct2, pKpn47-Ct1/2, p11935-Ct1/2, pQD1501-Ct1, p12085-Ct1, pA2293-Ct2, p397108-Ct2, p427113-Ct1/2, and pA1763-Ct2 were submitted to GenBank under the accession numbers MF344572 to MF344574, MN310369, MN310375, MN310377, MN310378, MH917284, MH917285, and MG764552, respectively.

## Results

### Overview of Sequenced IncC Plasmids

**FIGURE 1:**
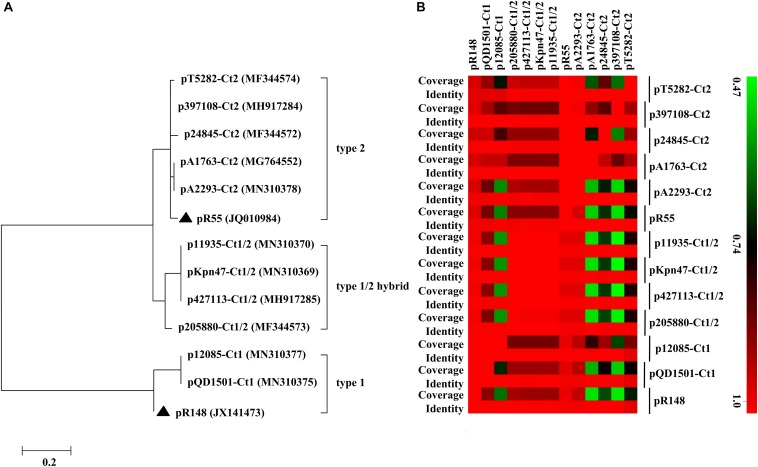
Evolutionary relationships of IncC plasmids. **(A)** A maximum-likelihood phylogenetic tree. The tree is constructed from recombination-free SNPs of core backbone regions. The degree of support (percentage) for each cluster of associated taxa, as determined by bootstrap analysis, is shown next to each branch. The bar corresponds to the scale of sequence divergence. Triangles indicate the references for type 1 and type 2 IncC plasmids. **(B)** A heatmap of pairwise comparison of plasmid backbone sequences. The original BLAST coverage and nucleotide identity values are shown in Supplementary Table S2.

Of the 11 IncC plasmids fully sequenced in this work (Table 1 and Figure 1), two and five could be assigned into type 1 and type 2, respectively, based on the presence or absence of *orf1832*/*orf1847*, *rhs1*/*rhs2*, i1, and i2, while the remaining four, containing *orf1832* and *rhs1* (characteristic of type 1) plus i2 (characteristic of type 2), were recognized as type 1/2 hybrid. A total of 597 core SNPs (among them 69 were recombination-free) were identified from the backbone regions of these 11 plasmids together with type 1 reference pR148 and type 2 reference pR55. As shown in the phylogenetic tree (Figure 1A) constructed from the 69 recombination-free SNPs, these 13 plasmids could be clustered into three separate clades, corresponding to the above three subtypes. A pairwise sequence comparison using *BLASTN* showed that these 13 plasmids displayed >99% nucleotide identity across ≥50% of their backbone sequences within each of subgroup IncC plasmids, and had >97% nucleotide identity across ≥47% of their backbone sequences between different subgroups (Figure 1B and Supplementary Table S2). The above results confirmed a parallel diversification and evolution of type 1, type 2, and type 1/2-hybrid IncC plasmids. These 13 plasmids varied in size from about 90 kb to nearly 206 kb with variation in the number of predicted ORFs from 118 to 246, and each plasmid was dissected into the IncC backbone regions and the accessory modules, which were defined as acquired DNA regions associated and bordered with mobile elements (Table 2 and Supplementary Figure S1). These 13 plasmids shared common IncC backbone genes *repA*, *parAB*, *tra2*, *dcm2*, *yacC*, *int*, *kfrA*, and *uvrD*.

**TABLE 1:** Type 1, 2, and 1/2-hybrid plasmids analyzed.

**IncC subtype**	**Plasmid**	**Accession number**	***orf1832/orf1847***	***rhs1/rhs2***	**i1**	**i2**	**Host bacterium**	**Location**	**References**
Type 1 reference	pR148	JX141473	*orf1832*	*rhs1*	*−*	*−*	*Aeromonas hydrophila*	Thailand	Del Castillo et al., 2013
Type 1	pQD1501-Ct1	MN310375	*orf1832*	*Δrhs1*	*−*	*−*	*Klebsiella quasipneumoniae*	China	This study
Type 1	p12085-Ct1	MN310377	-	*Δrhs1*	*−*	*−*	*K. pneumoniae*	China	This study
Type 2 reference	pR55	JQ010984	*orf1847*	*rhs2*	+	+	*K. pneumoniae*	France	Benoît et al., 2012
Type 2	pA2293-Ct2	MN310378	*orf1847*	*Δrhs2*	+	+	*K. pneumoniae*	China	This study
Type 2	pA1763-Ct2	MG764552	-	*Δrhs2*	+	+	*K. pneumoniae*	China	This study
Type 2	p24845-Ct2	MF344572	*orf1847*	*Δrhs2*	+	+	*Enterobacter hormaechei*	China	This study
Type 2	p397108-Ct2	MH917284	-	*rhs2*	+	*−*	*K. pneumoniae*	China	This study
Type 2	pT5282-Ct2	MF344574	*orf1847*	*rhs2*	−	+	*E. hormaechei*	China	This study
Type 1/2 hybrid	p205880-Ct1/2	MF344573	*orf1832*	*rhs1*	−	+	*K. pneumoniae*	China	This study
Type 1/2 hybrid	p427113-Ct1/2	MH917285	*orf1832*	*rhs1*	−	+	*K. pneumoniae*	China	This study
Type 1/2 hybrid	pKpn47-Ct1/2	MN310369	*orf1832*	*rhs1*	−	+	*K. pneumoniae*	China	This study
Type 1/2 hybrid	p11935-Ct1/2	MN310370	*orf1832*	*rhs1*	−	+	*K. pneumoniae*	China	This study

*Ct1, IncCtype 1; Ct2, IncCtype 2; Ct1/2, IncCtype 1/2 hybrid. Characteristics of type 1 IncC plasmids, presence of *orf1832* and *rhs1*; Characteristics of type 2 IncC plasmids, presence of *orf1847*, *rhs2*, i1, and i2. +, presence;−, absence.*

**TABLE 2:** Major features of analyzed IncC plasmids.

**Plasmid**	**Total length (bp)**	**Total number of ORFs**	**Mean G + C content (%)**	**Length of backbone (bp)**	**Mean G + C content of backbone (%)**	**Accessory modules**				
						**Resistance**				**Non-resistance**
						**ARI-A**	**ARI-B**	***bla*_CMY_-carrying region**	**Others**	
pR148	165,906	195	52.5	127,803	51.2	Tn*6358*	−	–	–	–
pQD1501–Ct1	132,407	161	51.9	113,444	51.2	+	−	Tn*6538b*	–	–
p12085–Ct1	90,252	122	51.7	79,675	51.1	−	−	*bla*_CMY–6_*–bla*_NDM–1_ region	*−*	*−*
pR55	170,810	203	53.0	129,210	51.2	*−*	+	*−*	Tn*6187*	*−*
pA2293–Ct2	181,726	205	51.0	124,622	51.1	*−*	+	*−*	*−*	IS*Kpn52*:IS*30*, IS*As25*, and IS*100kyp*
pA1763–Ct2	93,441	118	51.2	67,327	51.0	*−*	+	*−*	*bla*_CTX–M–3_ region	*−*
p24845–Ct2	167,619	192	49.9	89,202	51.0	*−*	*−*	*−*	MDR region	ΔTn*6292–*ΔIS*26–*IS*26* region
p397108–Ct2	105,977	133	52.5	64,665	50.7	*−*	+	*−*	Tn*6558*	IS*Kpn18*
pT5282–Ct2	152,215	173	52.8	94,867	51.2	*−*	+	*−*	*bla*_CTX–M–14_*–bla*_IMP–8_ region, and MDR region	IS*Ehe3*, and IS*Ehe3*
p205880–Ct1/2	153,373	182	52.3	117,240	51.2	Tn*6395*	+	Tn*6538a*	*−*	*−*
p427113–Ct1/2	205,674	246	53.3	116,457	51.3	+	+	Tn*6538a*	MDR region	*−*
pKpn47–Ct1/2	154,204	178	52.4	116,460	51.3	+	+	Tn*6538c*	*−*	IS*Kpn18*, IS*1R*, and IS*Kpn18*
p11935–Ct1/2	154,204	181	52.5	116,459	51.3	+	+	Tn*6538c*	*−*	IS*Kpn18*, IS*1R*, and IS*Kpn18*

*+, presence ARI-A or ARI-B, but not identified as an intact transposon; –, absence.*

### Major Modular Differences in Backbone and Accessory Regions

**FIGURE 2:**
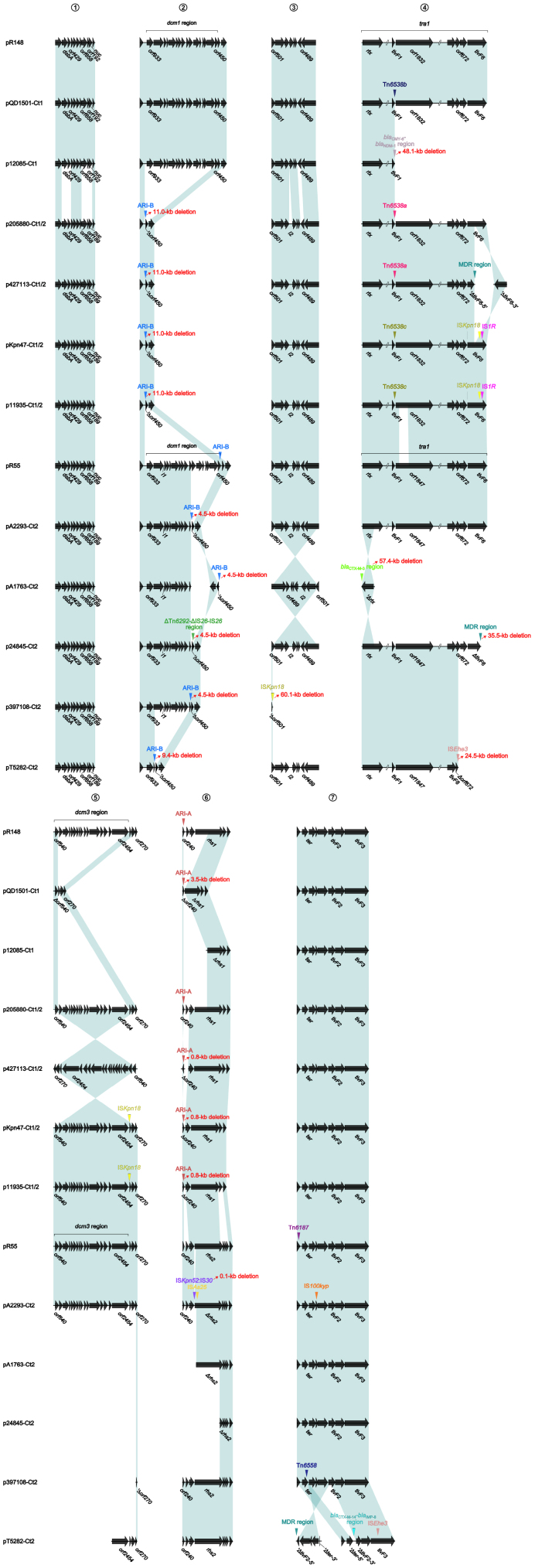
Major modular differences among IncC plasmids. Genes are denoted by arrows. Genes, mobile elements, and other features are colored based on function classification. Shading denotes regions of homology (≥95% nucleotide identity).

At least seven major modular differences were identified across the whole plasmid sequences (Figure 2 and Supplementary Figure S2). First, two small regions (*dsbA–orf429*, and *orf858–orf192/orf189–nuc*) displayed <95% nucleotide identity between all the three type 1 plasmids and all the 10 type 1/2-hybrid and type 2 plasmids, for which a gene substitution (*orf192* in the former three, and *orf189* in the later ten) occurred. Second, the ARI-B island or the ΔTn*6292–*ΔIS*26–*IS*26* region was inserted into the backbone *dcm1* region of all the 10 type 1/2-hybrid and type 2 plasmids, leading to a 4.5- to 11.0-kb deletion within the *dcm1* region of these 10 plasmids expect for pR55. Third, the three type 1 plasmids had a backbone gene *orf489* showing <90% nucleotide identity to all the other plasmids; IS*Kpn18* was inserted at a site within the backbone gene *orf501* in p397108-Ct2, resulting in a 60.1-kb deletion (containing the whole *tra1* region and the *dcm3* region). Fourth, compared to the prototype *tra1* region as observed in pR148 and additional two plasmids, various insertions of accessory modules were found in the other plasmids: (i) Tn*6538a* or Tn*6538b* or Tn*6538c* or the *bla*_CMY–__6_*–bla*_NDM–__1_ region was inserted downstream of *tivF1* of all the seven type 1 and type 1/2-hybrid plasmids except for pR148, resulting in a 48.1-kb deletion in p12085-Ct1; (ii) the insertion of IS*Ehe3* into *orf672* of pT5282-Ct2 or that of *bla*_CTX–M–__3_ region into *rlx* of pA1763-Ct2 led to a 24.5-kb or 57.4-kb deletion, respectively; and (iii) IS*Kpn18* plus IS*1R* was inserted into *tivF6* of pKpn47-Ct1/2 or p11935-Ct1/2, while the MDR (multidrug resistance) region was inserted into the same gene of p427113-Ct1/2 or p24845-Ct2, leading to a 35.5-kb deletion in p24845-Ct2. Fifth, the backbone *dcm3* region was completely or partially lost in five plasmids, which was caused by accessory module insertion or other unknown reasons. Sixth, the ARI-A island was inserted into the backbone gene *orf240* of all the seven type 1 and type 1/2-hybrid plasmids except for p12085-Ct1, while IS*Kpn52*:IS*30* plus IS*As25* was inserted into a type 2 plasmid pA2293-Ct2. Seventh, Tn*6187*, IS*100kyp* and Tn*6558* were inserted at different sites around the backbone *ter* gene of pR55, pA2293-Ct2, and p397108-Ct2, respectively; additionally, three separate insertions of the MDR region, the *bla*_CTX–M–__14_*–bla*_IMP–__8_ region, and the IS*Ehe3* element into the backbone *ter–*to*–tivF3* region made its segmentation in pT5282-Ct2.

### ARI-A and ARI-B Islands

**FIGURE 3:**
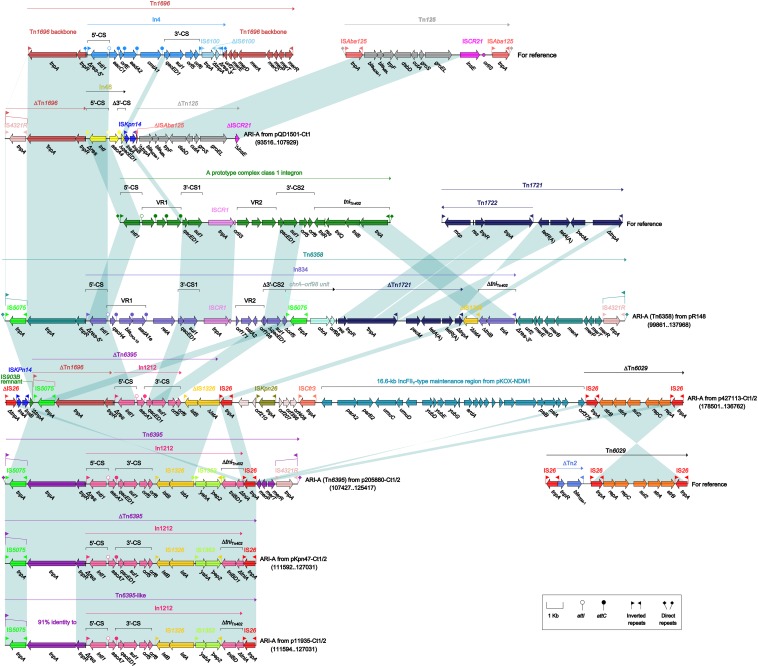
Organization of ARI-A islands and comparison to related regions. Genes are denoted by arrows. Genes, mobile elements, and other features are colored based on their functional classification. Shading denotes regions of homology (nucleotide identity ≥95%). Numbers in brackets indicate nucleotide positions within corresponding plasmids. The accession numbers of Tn*1696* (Partridge et al., 2001), Tn*125* (Poirel et al., 2012), Tn*1721* (Allmeier et al., 1992), and Tn*6029* (Cain et al., 2010) for reference are U12338, JN872328, X61367, and HQ840942, respectively.

As found in all the seven type 1 and type 1/2-hybrid plasmids except for p12085-Ct1 (type 1), the ARI-A islands (Figure 3) were identified as Tn*1696* (Partridge et al., 2001) derivatives. The Tn*1696* derivatives in pR148 and p205880-Ct1/2 were recognized as intact unit transposons Tn*6358* (Ma et al., 2017) and Tn*6395*, respectively, which differed from Tn*1696* mainly by insertion of In834 (Ma et al., 2017) and In1212 instead of In4 into a primary *tnpAR*–*mer* structure. The Tn*1696* derivatives in the other four plasmids could not be discriminated as intact transposons due to 3′-terminal truncations, which were in some cases accompanied with insertion of additional resistance modules: *bla*_NDM–__1_-carrying ΔTn*125* (Poirel et al., 2012) in pQD1501-Ct1 and *strAB*-carrying ΔTn*6029* (Cain et al., 2010) in p427113-Ct1/2.

**FIGURE 4:**
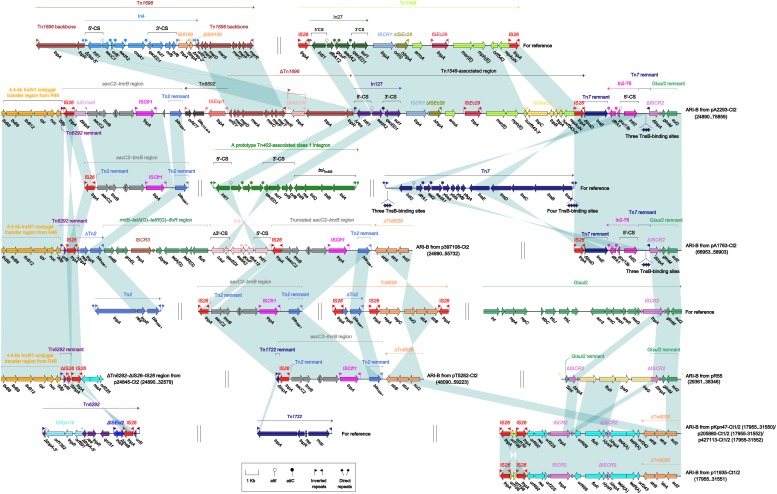
Organization of ARI-B islands and comparison to related regions. Genes are denoted by arrows. Genes, mobile elements, and other features are colored based on their functional classification. Shading denotes regions of homology (nucleotide identity ≥95%). Numbers in brackets indicate nucleotide positions within corresponding plasmids. The accession numbers of Tn*1696* (Partridge et al., 2001), Tn*1548* (Galimand et al., 2005), *aacC2*–*tmrB* region (Partridge et al., 2012), Tn*7* (see Zhan et al., 2018, for gene organization), Tn*2* (Bailey et al., 2011), Tn*6029* (Cain et al., 2010), GI*sul2* (Nigro and Hall, 2011), Tn*6292* (Wei et al., 2016), and Tn*1722* (Allmeier et al., 1992) for reference are U12338, AF550415, JX101693, KX117211, HM749967, HQ840942, AE014073, KU886034, and X61367, respectively.

The ARI-B islands (Figure 4) were found in all the 10 type 2 and type 1/2-hybrid plasmids except for p24845-Ct2 (type 2), and all of them contained a *sul2* gene that was originated from either GI*sul2* (Nigro and Hall, 2011) or Tn*6029* (Cain et al., 2010), because various *sul2*-carrying remnants of GI*sul2* or Tn*6029* were found in these ARI-B islands. In general, these ARI-B islands acquired dramatically different collection of resistance modules, which contained genes resistance to older (such as *dfrA*, *strAB*, *tetA*, and *floR*) and newer (such as *bla*_CTX–M_, *armA*, *msr*, and *mph*) antibiotics, and thus varied considerably in size with a highly mosaic nature. The ΔTn*6292–*ΔIS*26–*IS*26* region (Figure 4), which did not contain any of resistance genes, was inserted at the site specific for ARI-B insertion.

### Other Accessory Resistance Modules

**FIGURE 5:**
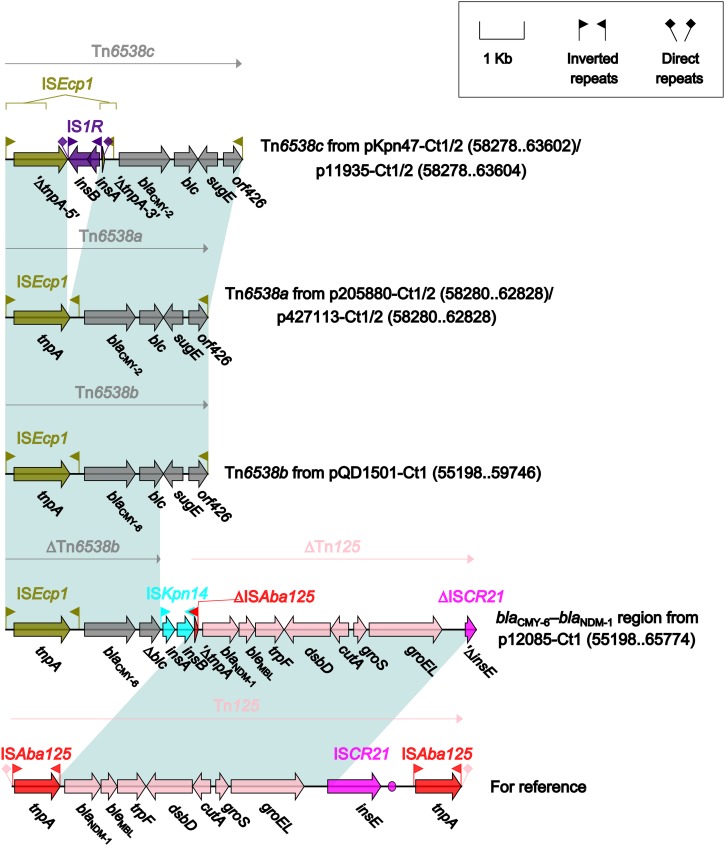
Organization of *bla*_CMY_-carrying regions and comparison to related regions. Genes are denoted by arrows. Genes, mobile elements and other features are colored based on their functional classification. Shading denotes regions of homology (nucleotide identity ≥95%). Numbers in brackets indicate nucleotide positions within corresponding plasmids. The accession number of Tn*125* (Poirel et al., 2012) for reference is JN872328.

Four different *bla*_CMY_-carrying regions (Figure 5) were found in all the seven type 1 and type 1/2-hybrid plasmids except for pR148 and were manifested as three intact IS*Ecp1*-based transposition units Tn*6538a* (p205880-Ct1/2 and p427113-Ct1/2), Tn*6538b* (pQD1501-Ct1), and Tn*6538c* (pKpn47-Ct1/2 and p11935-Ct1/2), and the *bla*_CMY–__6_*–bla*_NDM–__1_ region (p12085-Ct1). Tn*6538a*, Tn*6538b*, and Tn*6538c* were highly similar to one another but differed by substitution of *bla*_CMY–__2_ with *bla*_CMY–__6_ or by insertion of IS*1R* into IS*Ecp1*. The *bla*_CMY–__6_*–bla*_NDM–__1_ region was a combination of ΔTn*6538b* carrying *bla*_CMY–__6_, IS*Kpn14*, and ΔTn*125* containing *bla*_NDM–__1_.

**FIGURE 6:**
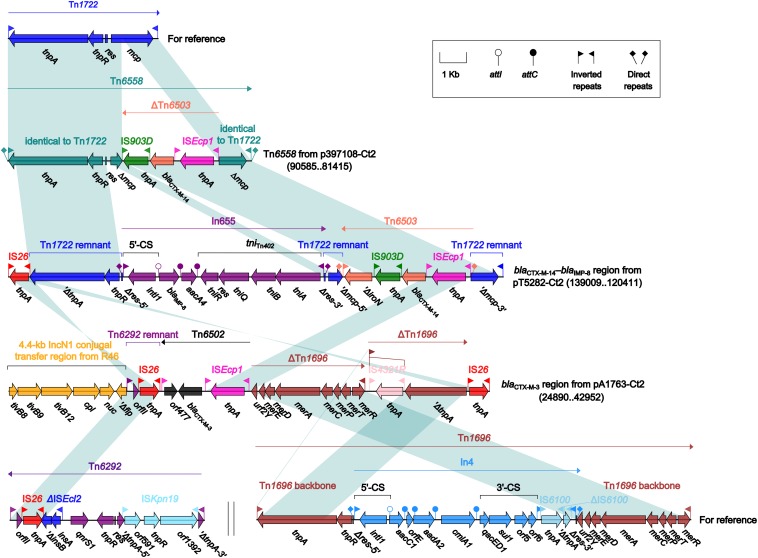
Organization of *bla*_CTX–M_-carrying regions and comparison to related regions. Genes are denoted by arrows. Genes, mobile elements and other features are colored based on their functional classification. Shading denotes regions of homology (nucleotide identity ≥95%). Numbers in brackets indicate nucleotide positions within corresponding plasmids. The accession numbers of Tn*1722* (Allmeier et al., 1992), Tn*6292* (Wei et al., 2016) and Tn*1696* (Partridge et al., 2001) for reference are X61367, KU886034 and U12338, respectively.

Four different *bla*_CTX–M_-carrying regions were found in four type 2 plasmids: Tn*6558* (p397108-Ct2), the *bla*_CTX–M–__14_*–bla*_IMP–__8_ region (pT5282-Ct2), and the *bla*_CTX–M–__3_ region (pA1763-Ct2) (Figure 6), as well as the ARI-B island (pA2293-Ct2) (Figure 4). Insertion of a truncated version of Tn*6503*, which was an IS*Ecp1*-based, *bla*_CTX–M–__14_-carrying transposition unit (Feng et al., 2015), into the cryptic unit transposon Tn*1722* generated Tn*6558*, while the *bla*_CTX–M–__14_*–bla*_IMP–__8_ region was resulted from three separate insertions of Tn*6503*, In655 (carrying *bla*_IMP–__8_), and IS*26* into Tn*1722*. The *bla*_CTX–M–__3_ region in pA1763-Ct2 was highly similar to a partial region of the ARI-B island in pA2293-Ct2, and both carried the IS*Ecp1*-based *bla*_CTX–M–__3_-carrying transposition unit Tn*6502* (Liang et al., 2014).

**FIGURE 7:**
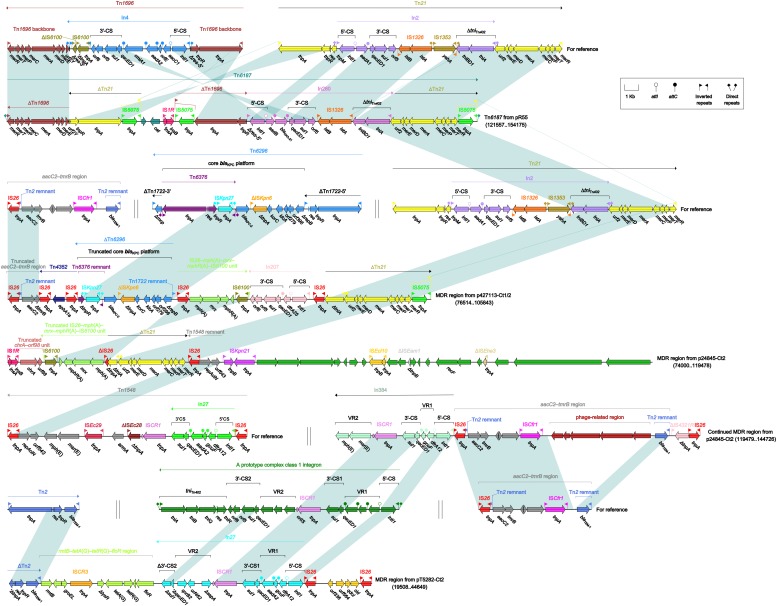
Organization of MDR regions and comparison to related regions. Genes are denoted by arrows. Genes, mobile elements and other features are colored based on their functional classification. Shading denotes regions of homology (nucleotide identity ≥95%). Numbers in brackets indicate nucleotide positions within corresponding plasmids. The accession number of Tn*1696* (Partridge et al., 2001), Tn*21* (Partridge et al., 2001), Tn*1548* (Galimand et al., 2005), Tn*2* (Bailey et al., 2011), *aacC2*–*tmrB* region (Partridge et al., 2012) and Tn*6296* (Jiang et al., 2010) for reference are U12338, AF071413, AF550415, HM749967, JX101693 and FJ628167, respectively.

Four different MDR regions (Figure 7) were found in four plasmids: Tn*6187* (Benoît et al., 2012) in pR55, and three MDR regions in p427113-Ct1/2, pT5282-Ct2, and p24845-Ct2. As similar to the above ARI-B islands, different MDR regions were assembled from different collections of resistance units [especially including ΔTn*6296* carrying *bla*_KPC–__2_, IS*26*–*mph*(A)–*mrx*–*mphR*(A)–IS*6100* unit, In207, In384, and *rmtB*–*tetA*(G)–*tetR*(G)–*floR* region] via homologous or non-homologous recombination.

### Transferability and Antimicrobial Susceptibility

Plasmids pQD1501-Ct1, pA2293-Ct2, and p205880-Ct1/2 were arbitrarily selected as the representatives of type 1, type 2, and type 1/2 hybrid, respectively, and could be transferred from the wild-type isolates into J53 or EC600 through conjugation, generating *E*. *coli* transconjugants QD1501-NDM-J53, A2293-CTXM-J53, and 205880-CMY-EC600, respectively. The self-transferable nature was consistent with the presence of two complete sets (*tra1* and *tra2*) of conjugal transfer genes in each plasmid, although Tn*6538a* or Tn*6538b* was inserted at an intergenic site in *tra1* of p205880-Ct1/2 or pQD1501-Ct1, respectively. All the three wild-type isolates QD1501, A2293, and 205880 and one transconjugant QD1501-NDM-J53 were resistant to imipenem with minimum inhibitory concentration (MIC) values ≥16 (due to production of NDM or KPC enzyme) but the other two transconjugants A2293-CTXM-J53 and 205880-CMY-EC600 remained susceptible to imipenem. All the above strains were resistant to cefuroxime with MIC values ≥32 (due to production of NDM or CTX-M or CMY enzyme).

## Discussion

**FIGURE 8:**
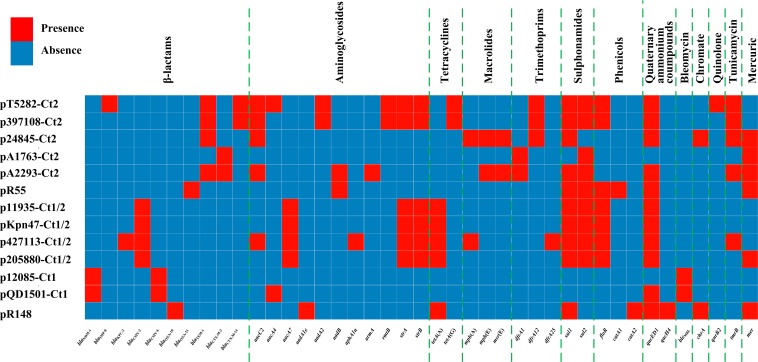
A heatmap of prevalence of resistance genes in IncC plasmids. The original data are shown in Supplementary Table S3.

Comparative genomics analysis of the collection of IncC plasmids (*n* = 13 in this study) provides a deeper understanding of diversification of IncC plasmids. Different IncC plasmids have evolved to acquire very different profiles of accessory modules and thus different collections of resistance genes (Figure 8). Insertion of accessory modules is often accompanied by deletion of surrounding backbone regions. Type 1/2-hybrid IncC plasmids most likely emerged from homologous recombination between type 1 and type 2 plasmids. In addition to using the presence or absence of signature sequences *orf1832*/*orf1847*, *rhs1*/*rhs2*, i1, and i2, a phylogenetic analysis using recombination-free SNPs of core backbone regions can steadily discriminate type 1/2 hybrid from type 1 and type 2. As shown previously (Ma et al., 2017) and herein, additional resistance islands (e.g., the MDR regions and the *bla*_CTX–M_-carrying regions in this study) can be found in type 1, type 2, and type 1/2-hybrid plasmids.

The IncC reference plasmids pR148 (166 kb in length, 195 ORFs) and pR55 (171 kb in length, 203 ORFs) had the most complete backbones for type 1 and type 2, respectively. By contrast, the type 1 plasmid p12085-Ct1 and the type 2 plasmids pA1763-Ct2 and p397108-Ct2 harbored extremely simple backbones due to deletion of large backbone regions. In addition to insertion of accessory modules and deletion of backbone regions, large inversion (such as those in p427113-Ct1/2 and pA1763-Ct2) and translocation (such as those in pT5282-Ct2) events occurred within backbone regions during genomic diversification of IncC plasmids (Ma et al., 2017; Supplementary Figure S2).

The carbapenemase genes *bla*_NDM–__1_, *bla*_KPC–__2_, and *bla*_IMP–__8_ were identified in pQD1501-Ct1 and p12085-Ct1, p427113-Ct1/2, and pT5282-Ct2, respectively. This is the first report of a *bla*_IMP–__8_-carrying IncC plasmid. This study presents three novel mobile elements [firstly identified in this study, including Tn*6395* (p205880-Ct1/2), In2-76 (pA1763-Ct2), and IS*Ecl10* (p24845-Ct2)] and additionally six newly named mobile elements [firstly designated in this study but with previously determined sequences, including Tn*6538a* (p205880-Ct1/2 and p427113-Ct1/2), Tn*6538b* (pQD1501-Ct1), Tn*6538c* (pKpn47-Ct1/2 and p11935-Ct1/2), Tn*6502* (pA2293-Ct2 and pA1763-Ct2), Tn*6503* (pT5282-Ct2), and IS*Kpn52* (pA2293-Ct2)].

## Data Availability Statement

The datasets generated for this study can be found in the complete nucleotide sequences of plasmids p24845-Ct2, p205880-Ct1/2, pT5282-Ct2, pKpn47-Ct1/2, p11935-Ct1/2, pQD1501-Ct1, p12085-Ct1, pA2293-Ct2, p397108-Ct2, p427113-Ct1/2, and pA1763-Ct2 were submitted to GenBank under the accession numbers MF344572 to MF344574, MN310369, MN310370, MN310375, MN310377, MN310378, MH917284, MH917285, and MG764552, respectively.

## Ethics Statement

This study uses the clinical bacterial isolates obtained from the Chinese public hospitals as listed in Supplementary Table S1. The local legislation did not require the study to be reviewed or approved by an ethics committee, because the bacterial isolates involved in this study were part of the routine hospital laboratory procedures. The research involving biohazards and all related procedures were approved by the Biosafety Committee of the Beijing Institute of Microbiology and Epidemiology.

## Author Contributions

ED and DZ conceived the study and designed the experimental procedures. QC, XJ, ZY, YX, LH, and HG performed the experiments. QC, WL, XJ, DZ, and ED analyzed the data. WY, HY, YZ, and XZ provided the reagents and materials. DZ, QC, and ED wrote the manuscript.

## Conflict of Interest

The authors declare that the research was conducted in the absence of any commercial or financial relationships that could be construed as a potential conflict of interest.

## Footnotes

1 https://github.com/ruanjue/smartdenovo

2 https://inkscape.org/en/

## References

[B1] Allmeier H. Cresnar B. Greck M. Schmitt R. (1992). Complete nucleotide sequence of Tn*1721*: gene organization and a novel gene product with features of a chemotaxis protein. *Gene* 111 11–20. 10.1016/0378-1119(92)90597-i 1312499

[B2] Ambrose S. J. Harmer C. J. Hall R. M. (2018). Compatibility and entry exclusion of IncA and IncC plasmids revisited: IncA and IncC plasmids are compatible. *Plasmid* 96-97 7–12. 10.1016/j.plasmid.2018.02.002 29486211

[B3] Bailey J. K. Pinyon J. L. Anantham S. Hall R. M. (2011). Distribution of the *bla*_TEM_ gene and *bla*_TEM_-containing transposons in commensal *Escherichia coli*. *J. Antimicrob. Chemother.* 66 745–751. 10.1093/jac/dkq529 21393132

[B4] Benoît D. David B. Gregory D. Karine P. Axel C. Mulvey M. R. (2012). Complete nucleotide sequence of the multidrug resistance IncA/C plasmid pR55 from *Klebsiella pneumoniae* isolated in 1969. *J. Antimicrob. Chemother.* 67 2354–2360. 10.1093/jac/dks251 22773739

[B5] Boetzer M. Pirovano W. (2012). Toward almost closed genomes with gapfiller. *Genome Biol.* 13:R56. 10.1186/gb-2012-13-6-r56 22731987 PMC3446322

[B6] Bolger A. M. Lohse M. Usadel B. (2014). Trimmomatic: a flexible trimmer for illumina sequence data. *Bioinformatics* 30 2114–2120. 10.1093/bioinformatics/btu170 24695404 PMC4103590

[B7] Boratyn G. M. Camacho C. Cooper P. S. Coulouris G. Fong A. Ma N. (2013). BLAST: a more efficient report with usability improvements. *Nucleic Acids Res.* 41 W29–W33. 10.1093/nar/gkt282 23609542 PMC3692093

[B8] Boutet E. Lieberherr D. Tognolli M. Schneider M. Bansal P. Bridge A. J. (2016). UniProtKB/Swiss-Prot, the manually annotated section of the uniprot knowledgebase: how to use the entry view. *Methods Mol. Biol.* 1374 23–54. 10.1007/978-1-4939-3167-5_2 26519399

[B9] Brettin T. Davis J. J. Disz T. Edwards R. A. Gerdes S. Olsen G. J. (2015). RASTtk: a modular and extensible implementation of the rast algorithm for building custom annotation pipelines and annotating batches of genomes. *Sci. Rep.* 5:8365. 10.1038/srep08365 25666585 PMC4322359

[B10] Cain A. Liu X. Djordjevic S. Hall R. (2010). Transposons related to Tn*1696* in IncHI2 plasmids in multiply antibiotic resistant *Salmonella enterica Serovar typhimurium* from Australian animals. *Microb. Drug Resist.* 16 197–202. 10.1089/mdr.2010.0042 20701539

[B11] CLSI (2017). *Performance Standards for Antimicrobial Susceptibility Testing*, 27th Edn Wayne: CLSI.

[B12] Del Castillo C. S. Jun-Ichi H. Ho-Bin J. Seong-Won N. Tae-Sung J. Janenuj W. (2013). Comparative sequence analysis of a multidrug-resistant plasmid from aeromonas hydrophila. *Antimicrob. Agents Chemother.* 57 120–129. 10.1128/AAC.01239-12 23070174 PMC3535917

[B13] Didelot X. Wilson D. J. (2015). ClonalFrameML: efficient inference of recombination in whole bacterial genomes. *PLoS Comput. Biol.* 11:e1004041. 10.1371/journal.pcbi.1004041 25675341 PMC4326465

[B14] Edgar R. C. (2004). MUSCLE: multiple sequence alignment with high accuracy and high throughput. *Nucleic Acids Res.* 32 1792–1797. 10.1093/nar/gkh340 15034147 PMC390337

[B15] Feng J. Qiu Y. Yin Z. Chen W. Yang H. Yang W. (2015). Coexistence of a novel KPC-2-encoding MDR plasmid and an NDM-1-encoding pNDM-HN380-like plasmid in a clinical isolate of *Citrobacter freundii*. *J Antimicrob. Chemother.* 70 2987–2991. 10.1093/jac/dkv232 26260129

[B16] Fricke W. F. Welch T. J. Mcdermott P. F. Mammel M. K. Leclerc J. E. White D. G. (2009). Comparative genomics of the IncA/C multidrug resistance plasmid family. *J. Bacteriol.* 191 4750–4757. 10.1128/JB.00189-09 19482926 PMC2715731

[B17] Galimand M. Sabtcheva S. Courvalin P. Lambert T. (2005). Worldwide disseminated armA aminoglycoside resistance methylase gene is borne by composite transposon Tn*1548*. *Antimicrob. Agents Chemother.* 49 2949–2953. 10.1128/aac.49.7.2949-2953.2005 15980373 PMC1168633

[B18] Hackl T. Hedrich R. Schultz J. Forster F. (2014). Proovread: large-scale high-accuracy PacBio correction through iterative short read consensus. *Bioinformatics* 30 3004–3011. 10.1093/bioinformatics/btu392 25015988 PMC4609002

[B19] Harmer C. J. Hall R. M. (2014). pRMH760, a precursor of A/C? plasmids carrying *bla*_CMY_ and *bla*_*NDM*_ genes. *Microb. Drug Resist.* 20 416–423. 10.1089/mdr.2014.0012 24841670

[B20] Harmer C. J. Hall R. M. (2015). The A to Z of A/C plasmids. *Plasmid* 80 63–82. 10.1016/j.plasmid.2015.04.003 25910948

[B21] Harmer C. J. Hamidian M. Hall R. M. (2017). pIP40a, a type 1 IncC plasmid from 1969 carries the integrative element GI sul2 and a novel class II mercury resistance transposon. *Plasmid* 92 17–25. 10.1016/j.plasmid.2017.05.004 28577759

[B22] Jia B. Raphenya A. R. Alcock B. Waglechner N. Guo P. Tsang K. K. (2017). CARD 2017: expansion and model-centric curation of the comprehensive antibiotic resistance database. *Nucleic Acids Res.* 45 D566–D573. 10.1093/nar/gkw1004 27789705 PMC5210516

[B23] Jiang Y. Yu D. L. Wei Z. Q. Shen P. Zhou Z. H. Yu Y. S. (2010). Complete nucleotide sequence of *Klebsiella pneumoniae* multidrug resistance plasmid pKP048, carrying *bla*_*KPC–2*_, *bla*_*DHA–1*_, *qnrB4*, and *armA*. *Antimicrob. Agents Chemother.* 54 3967–3969. 10.1128/AAC.00137-10 20547789 PMC2934982

[B24] Kumar S. Stecher G. Tamura K. (2016). MEGA7: molecular evolutionary genetics analysis version 7.0 for bigger datasets. *Mol. Biol. Evol.* 33 1870–1874. 10.1093/molbev/msw054 27004904 PMC8210823

[B25] Kurtz S. Phillippy A. Delcher A. L. Smoot M. Shumway M. Antonescu C. (2004). Versatile and open software for comparing large genomes. *Genome Biol.* 5:R12. 14759262 10.1186/gb-2004-5-2-r12 PMC395750

[B26] Liang C. Hongyan H. Chavda K. D. Shulong Z. Renkun L. Hui L. (2014). Complete sequence of a KPC-producing IncN multidrug-resistant plasmid from an epidemic *Escherichia coli* sequence type 131 strain in China. *Antimicrob. Agents Chemother.* 58 2422–2425. 10.1128/AAC.02587-13 24395232 PMC4023777

[B27] Ma L. Yin Z. Zhang D. Zhan Z. Wang Q. Duan X. (2017). Comparative genomics of type 1 IncC plasmids from China. *Future Microbiol.* 12 1511–1522. 10.2217/fmb-2017-0072 29140102

[B28] Moura A. Soares M. Pereira C. Leitão N. Henriques I. Correia A. (2009). INTEGRALL: a database and search engine for integrons, integrases and gene cassettes. *Bioinformatics* 25 1096–1098. 10.1093/bioinformatics/btp105 19228805

[B29] Nederbragt A. J. (2014). On the middle ground between open source and commercial software - the case of the newbler program. *Genome Biol.* 15:113. 10.1186/gb4173 25180324 PMC4054848

[B30] Nigro S. J. Hall R. M. (2011). GI*sul2*, a genomic island carrying the *sul2* sulphonamide resistance gene and the small mobile element CR2 found in the *Enterobacter cloacae* subspecies *cloacae* type strain ATCC 13047 from 1890, *Shigella flexneri* ATCC 700930 from 1954 and *Acinetobacter baumannii* ATCC 17978 from 1951. *J. Antimicrob. Chemother.* 66 2175–2176. 10.1093/jac/dkr230 21653606

[B31] O’Leary N. A. Wright M. W. Rodney B. J. Stacy C. Diana H. Rich M. V. (2016). Reference sequence (RefSeq) database at NCBI: current status, taxonomic expansion, and functional annotation. *Nucleic Acids Res.* 44 D733–D745. 10.1093/nar/gkv1189 26553804 PMC4702849

[B32] Partridge S. Brown H. Stokes H. Hall R. (2001). Transposons Tn*1696* and Tn*21* and their integrons In4 and In2 have independent origins. *Antimicrob. Agents Chemother.* 45 1263–1270. 10.1128/aac.45.4.1263-1270.2001 11257044 PMC90453

[B33] Partridge S. R. Ginn A. N. Paulsen I. T. Iredell J. R. (2012). pEl1573 carrying *bla*_*IMP–4*_, from Sydney, Australia, is closely related to other IncL/M plasmids. *Antimicrob. Agents Chemother.* 56 6029–6032. 10.1128/AAC.01189-12 22926566 PMC3486572

[B34] Poirel L. Bonnin R. A. Boulanger A. Schrenzel J. Kaase M. Nordmann P. (2012). Tn*125*-related acquisition of *bla*_NDM_-like genes in *Acinetobacter baumannii*. *Antimicrob. Agents Chemother.* 56 1087–1089. 10.1128/AAC.05620-11 22143526 PMC3264265

[B35] Richter M. Rossello-Mora R. (2009). Shifting the genomic gold standard for the prokaryotic species definition. *Proc. Natl. Acad. Sci. U.S.A.* 106 19126–19131. 10.1073/pnas.0906412106 19855009 PMC2776425

[B36] Roberts A. P. Chandler M. Courvalin P. Guedon G. Mullany P. Pembroke T. (2008). Revised nomenclature for transposable genetic elements. *Plasmid* 60 167–173. 10.1016/j.plasmid.2008.08.001 18778731 PMC3836210

[B37] Saeed A. I. Sharov V. White J. Li J. Liang W. Bhagabati N. (2003). TM4: a free, open-source system for microarray data management and analysis. *Biotechniques* 34 374–378. 10.2144/03342mt01 12613259

[B38] Siguier P. Perochon J. Lestrade L. Mahillon J. Chandler M. (2006). ISfinder: the reference centre for bacterial insertion sequences. *Nucleic Acids Res.* 34 32–36. 16381877 10.1093/nar/gkj014 PMC1347377

[B39] Wei F. Zhou D. Qian W. Luo W. Zhang D. Qiang S. (2016). Dissemination of IMP-4-encoding pIMP-HZ1-related plasmids among *Klebsiella pneumoniae* and *Pseudomonas aeruginosa* in a Chinese teaching hospital. *Sci. Rep.* 6:33419. 10.1038/srep33419 27641711 PMC5027574

[B40] Zankari E. Hasman H. Cosentino S. Vestergaard M. Rasmussen S. Lund O. (2012). Identification of acquired antimicrobial resistance genes. *J. Antimicrob. Chemother.* 67 2640–2644. 10.1093/jac/dks261 22782487 PMC3468078

[B41] Zhan Z. Hu L. Jiang X. Zeng L. Feng J. Wu W. (2018). Plasmid and chromosomal integration of four novel *bla*_IMP_-carrying transposons from *Pseudomonas aeruginosa*, *Klebsiella pneumoniae* and an *Enterobacter* sp. *J. Antimicrob. Chemother.* 73 3005–3015. 10.1093/jac/dky288 30351436

