# Supplementary figures and images for: Type 1, 2, and 1/2-Hybrid IncC Plasmids From China
Source: Front Microbiol. 2019 Nov 15;10:2508. doi: 10.3389/fmicb.2019.02508 (PMC6872532; doi:10.3389/fmicb.2019.02508)

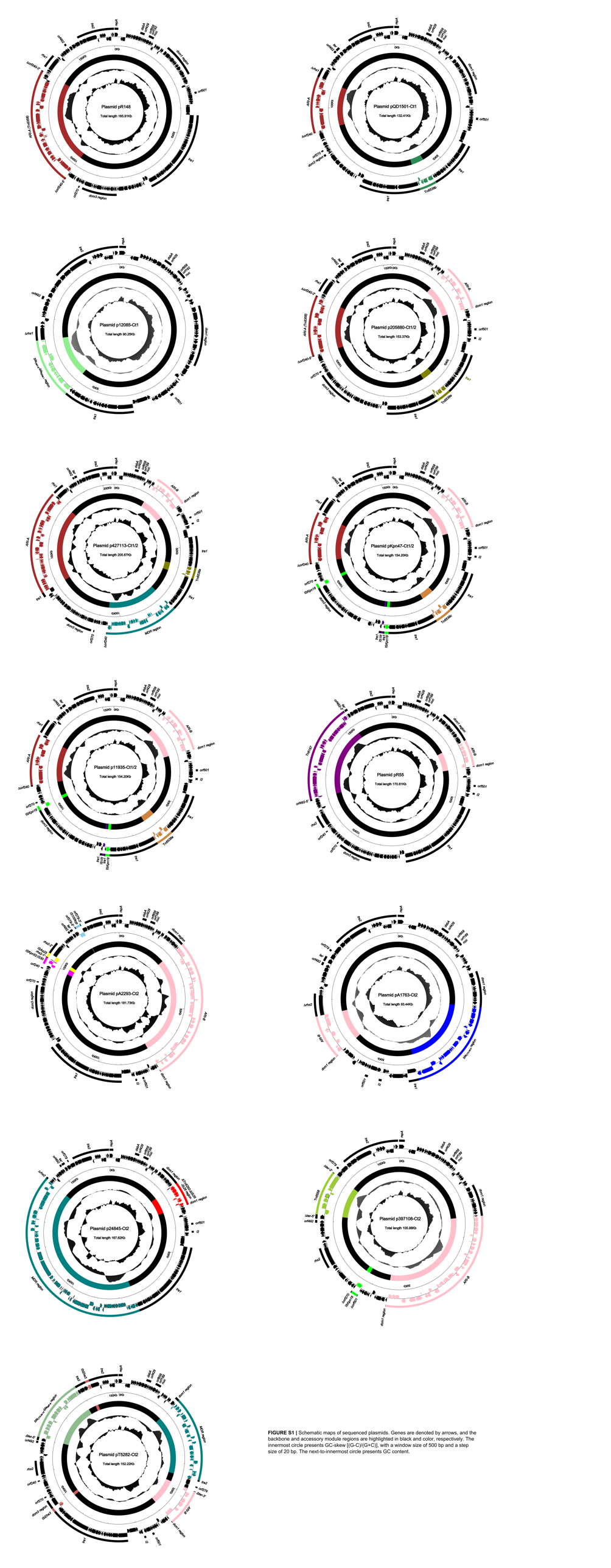

Supplement: Supplementary file 1 [file Image_1.tif]

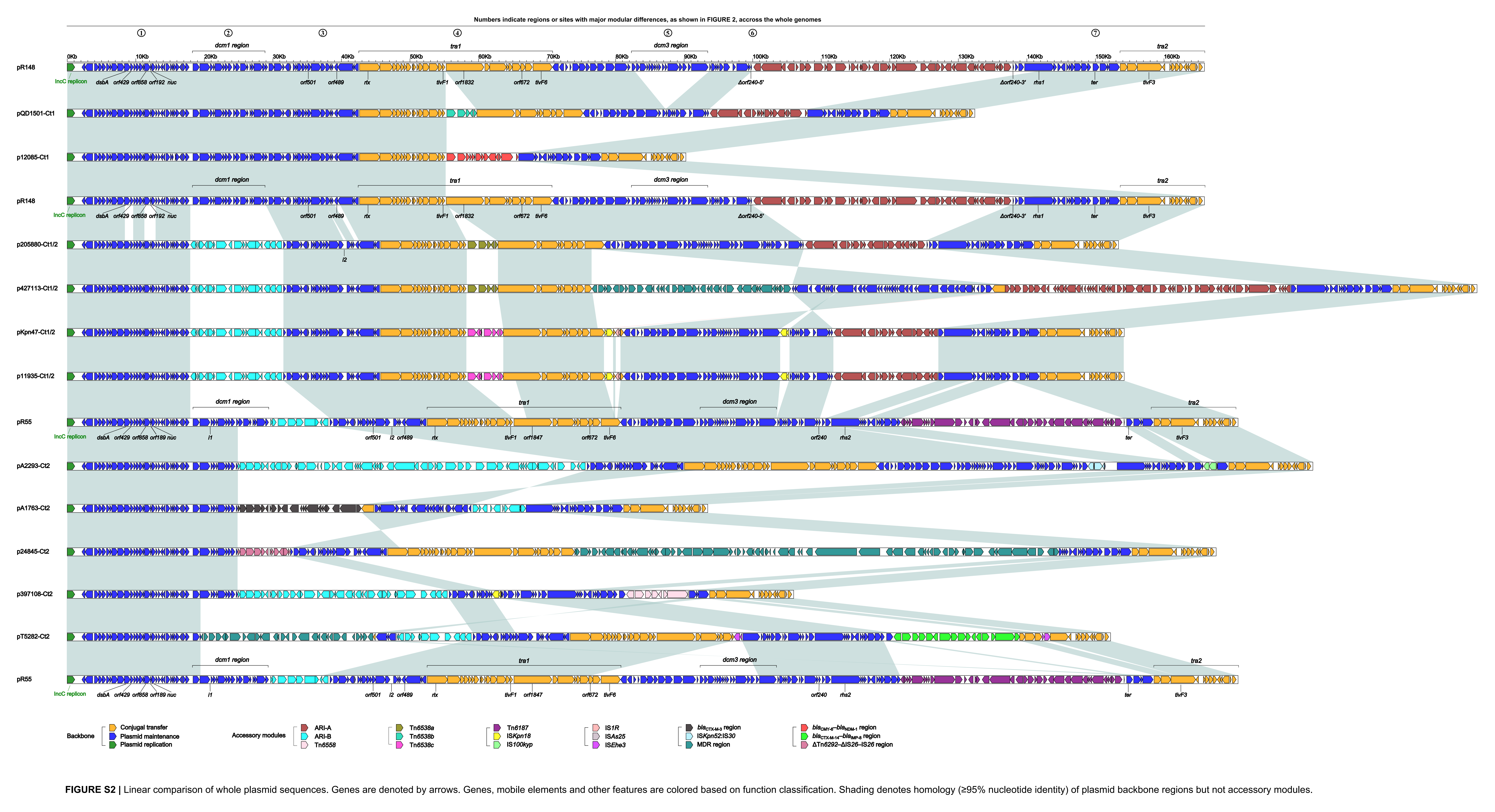

Supplement: Supplementary file 2 [file Image_2.tif]
