# Supplementary material for: Type 1, 2, and 1/2-Hybrid IncC Plasmids From China
Source: Front Microbiol. 2019 Nov 15;10:2508. doi: 10.3389/fmicb.2019.02508 (PMC6872532; doi:10.3389/fmicb.2019.02508)
Supplement: Supplementary file 5 [file Table_3.DOCX]

**TABLE S3 | Resistance genes in plasmids analyzed**

| **Plasmid** | **Resistance marker** | **Resistance phenotype** | **Nucleotide position** | **Region located** |
| --- | --- | --- | --- | --- |
| pR148 | *qacH4* | Quaternary ammonium compound resistance | 106328..106660 | ARI-A (Tn*6358*) |
|  | *bla*_OXA-10_ | β-lactam resistance | 106906..107706 |  |
|  | *aadA1e* | Aminoglycoside resistance | 107723..108514 |  |
|  | *qacED1* | Quaternary ammonium compound resistance | 110516..110863 |  |
|  | *sul1* | Sulphonamide resistance | 110857..111696 |  |
|  | *catA2* | Phenicol resistance | 114393..115034 |  |
|  | *∆qacED1* | Quaternary ammonium compound resistance | 115772..115987 |  |
|  | *sul1* | Sulphonamide resistance | 115981..116820 |  |
|  | *chrA* | Chromate resistance | 118635..119840 |  |
|  | *tetA*(A) | Tetracycline resistance | 125446..126645 |  |
|  | *mer* locus | Mercuric resistance | 132315..136582 |  |
| pQD1501-Ct1 | *bla*_CMY-6_ | β-lactam resistance | 56970..58115 | Tn*6538b* |
|  | *aacA4* | Aminoglycoside resistance | 99957..100511 | ARI-A |
|  | *∆qacED1* | Quaternary ammonium compound resistance | 100680..100856 |  |
|  | *bla*_NDM-1_ | β-lactam resistance | 101780..102592 |  |
|  | *ble*_MBL_ | Bleomycin resistance | 102596..102961 |  |
| p12085-Ct1 | *bla*_CMY-6_ | β-lactam resistance | 56970..58115 | *bla*_CMY-6_–*bla*_NDM-1_ region |
|  | *bla*_NDM-1_ | β-lactam resistance | 59625..60437 |  |
|  | *ble*_MBL_ | Bleomycin resistance | 60441..60806 |  |
| pR55 | *floR* | Phenicol resistance | 32207..33420 | ARI-B |
|  | *sul2* | Sulphonamide resistance | 37235..38050 |  |
|  | *mer*-1 locus | Mercuric resistance | 121595..125571 | Tn*6187* |
|  | *catA1* | Phenicol resistance | 131494..132153 |  |
|  | *aadB* | Aminoglycoside resistance | 139580..140113 |  |
|  | *bla*_OXA-21_ | β-lactam resistance | 140183..141010 |  |
|  | *qacED1* | Quaternary ammonium compound resistance | 141146..141493 |  |
|  | *sul1* | Sulphonamide resistance | 141487..142326 |  |
|  | *mer*-2 locus | Mercuric resistance | 148848..152810 |  |
| pA2293-Ct2 | *aacC2* | Aminoglycoside resistance | 31323..32183 | ARI-B |
|  | *tmrB* | Tunicamycin resistance | 32196..32738 |  |
|  | *bla*_TEM-1_ | β-lactam resistance | 37542..38402 |  |
|  | *bla*_CTX-M-3_ | β-lactam resistance | 39184..40059 |  |
|  | *mer* locus | Mercuric resistance | 42199..46175 |  |
|  | *aadA2* | Aminoglycoside resistance | 52702..53481 |  |
|  | *qacED1* | Quaternary ammonium compound resistance | 53645..53992 |  |
|  | *sul1* | Sulphonamide resistance | 53986..54825 |  |
|  | *armA* | Aminoglycoside resistance | 58170..58943 |  |
|  | *msr*(E) | Macrolide, Lincosamide and Streptogramin B resistance | 61242..62717 |  |
|  | *mph*(E) | Macrolide resistance | 62773..63657 |  |
|  | *dfrA1* | Trimethoprim resistance | 70476..70949 |  |
|  | *sul2* | Sulphonamide resistance | 74748..75563 |  |
| pA1763-Ct2 | *bla*_CTX-M-3_ | β-lactam resistance | 31262..32137 | *bla*_CTX-M-3_ region |
|  | *mer* locus | Mercuric resistance | 34277..38253 |  |
|  | *sul2* | Sulphonamide resistance | 59199..60014 | ARI-B |
|  | *dfrA1* | Trimethoprim resistance | 63813..64286 |  |
| p24845-Ct2 | *chrA* | Chromate resistance | 74903..76108 | MDR region |
|  | *mph*(A) | Macrolide resistance | 79727..80632 |  |
|  | *mer* locus | Mercuric resistance | 82374..86336 |  |
|  | *mph*(E) | Macrolide resistance | 119479..120363 |  |
|  | *msr*(E) | Macrolide, Lincosamide and Streptogramin B resistance | 120419..121894 |  |
|  | *sul1* | Sulphonamide resistance | 124096..124935 |  |
|  | *qacED1* | Quaternary ammonium compound resistance | 124929..125276 |  |
|  | *dfrA12* | Trimethoprim resistance | 125783..126280 |  |
|  | *aacC2* | Aminoglycoside resistance | 128650..129510 |  |
|  | *tmrB* | Tunicamycin resistance | 129523..130065 |  |
|  | *bla*_TEM-1_ | β-lactam resistance | 141620..142480 |  |
| p397108-Ct2 | *bla*_TEM-1_ | β-lactam resistance | 31568..32428 | ARI-B |
|  | *rmtB* | Aminoglycoside resistance | 32598..33353 |  |
|  | *tetA*(G) | Tetracycline resistance | 37058..38233 |  |
|  | *floR* | Phenicol resistance | 39170..40384 |  |
|  | *∆sul* | Sulphonamide resistance | 40601..41572 |  |
|  | *qacED1* | Quaternary ammonium compound resistance | 41566..41913 |  |
|  | *aadA2* | Aminoglycoside resistance | 42077..42856 |  |
|  | *dfrA12* | Trimethoprim resistance | 43276..43773 |  |
|  | *∆aacC2* | Aminoglycoside resistance | 45979..46674 |  |
|  | *tmrB* | Tunicamycin resistance | 46687..47229 |  |
|  | *bla*_TEM-1_ | β-lactam resistance | 51646..52506 |  |
|  | *strB* | Aminoglycoside resistance | 52921..53757 |  |
|  | *strA* | Aminoglycoside resistance | 53757..54560 |  |
|  | *sul2* | Sulphonamide resistance | 54621..55436 |  |
|  | *bla*_CTX-M-14_ | β-lactam resistance | 84353..85228 | Tn*6558* |
| pT5282-Ct2 | *bla*_TEM-1_ | β-lactam resistance | 20699..21559 | MDR region |
|  | *rmtB* | Aminoglycoside resistance | 21729..22484 |  |
|  | *tetA*(G) | Tetracycline resistance | 26189..27364 |  |
|  | *floR* | Phenicol resistance | 28301..29515 |  |
|  | *Δsul1* | Sulphonamide resistance | 30077..30703 |  |
|  | *∆qacED1* | Quaternary ammonium compound resistance | 30697..30901 |  |
|  | *qnrB2* | Quinolone resistance | 31194..31838 |  |
|  | *sul1* | Sulphonamide resistance | 35679..36518 |  |
|  | *qacED1* | Quaternary ammonium compound resistance | 36512..36859 |  |
|  | *aadA2* | Aminoglycoside resistance | 37023..37802 |  |
|  | *dfrA12* | Trimethoprim resistance | 38222..38719 |  |
|  | *aacC2* | Aminoglycoside resistance | 49305..50165 | ARI-B |
|  | *tmrB* | Tunicamycin resistance | 50178..50720 |  |
|  | *bla*_TEM-1_ | β-lactam resistance | 55137..55997 |  |
|  | *strB* | Aminoglycoside resistance | 56412..57248 |  |
|  | *strA* | Aminoglycoside resistance | 57248..58051 |  |
|  | *sul2* | Sulphonamide resistance | 58112..58927 |  |
|  | *bla*_CTX-M-14_ | β-lactam resistance | 123344..124219 | *bla*_CTX-M-14_–*bla*_IMP-8_ region |
|  | *aacA4* | Aminoglycoside resistance | 131941..132495 |  |
|  | *bla*_IMP-8_ | β-lactam resistance | 132594..133334 |  |
| p205880-Ct1/2 | *floR* | Phenicol resistance | 24392..25606 | ARI-B |
|  | *tetA*(A) | Tetracycline resistance | 26206..27480 |  |
|  | *strB* | Aminoglycoside resistance | 28741..29577 |  |
|  | *strA* | Aminoglycoside resistance | 29577..30380 |  |
|  | *sul2* | Sulphonamide resistance | 30441..31256 |  |
|  | *bla*_CMY-2_ | β-lactam resistance | 60052..61197 | Tn*6538a* |
|  | *aacA7* | Aminoglycoside resistance | 113809..114267 | ARI-A (Tn*6395*) |
|  | *qacED1* | Quaternary ammonium compound resistance | 114397..114744 |  |
|  | *sul1* | Sulphonamide resistance | 114738..115577 |  |
|  | *mer* locus | Mercuric resistance | 122884..124031 |  |
| p427113-Ct1/2 | *floR* | Phenicol resistance | 24392..25606 | ARI-B |
|  | *tetA*(A) | Tetracycline resistance | 26206..27480 |  |
|  | *strB* | Aminoglycoside resistance | 28741..29577 |  |
|  | *strA* | Aminoglycoside resistance | 29577..30380 |  |
|  | *sul2* | Sulphonamide resistance | 30441..31256 |  |
|  | *bla*_CMY-2_ | β-lactam resistance | 60052..61197 | Tn*6538a* |
|  | *aacC2* | Aminoglycoside resistance | 77503..78363 | MDR region |
|  | *ΔtmrB* | Tunicamycin resistance | 78376..78720 |  |
|  | *aphA1a* | Aminoglycoside resistance | 79626..80441 |  |
|  | *bla*_KPC-2_ | β-lactam resistance | 83188..84069 |  |
|  | *mph*(A) | Macrolide resistance | 89126..90031 |  |
|  | *sul1* | Sulphonamide resistance | 94242..95081 |  |
|  | *qacED1* | Quaternary ammonium compound resistance | 95075..95422 |  |
|  | *dfrA25* | Trimethoprim resistance | 95606..96064 |  |
|  | *mer* locus | Mercuric resistance | 100521..104483 |  |
|  | *sul2* | Sulphonamide resistance | 139452..140267 | ARI-A |
|  | *strA* | Aminoglycoside resistance | 140328..141131 |  |
|  | *strB* | Aminoglycoside resistance | 141131..141967 |  |
|  | *sul1* | Sulphonamide resistance | 168604..169443 |  |
|  | *qacED1* | Quaternary ammonium compound resistance | 169437..169784 |  |
|  | *aacA7* | Aminoglycoside resistance | 169914..170372 |  |
| pKpn47-Ct1/2 | *floR* | Phenicol resistance | 24392..25606 | ARI-B |
|  | *tetA*(A) | Tetracycline resistance | 26206..27480 |  |
|  | *strB* | Aminoglycoside resistance | 28741..29577 |  |
|  | *strA* | Aminoglycoside resistance | 29577..30379 |  |
|  | *sul2* | Sulphonamide resistance | 30440..31254 |  |
|  | *bla*_CMY-2_ | β-lactam resistance | 60826..61971 | Tn*6538c* |
|  | *aacA7* | Aminoglycoside resistance | 117969..118427 | ARI-A |
|  | *qacED1* | Quaternary ammonium compound resistance | 118557..118904 |  |
|  | *sul1* | Sulphonamide resistance | 118898..119737 |  |
| p11935-Ct1/2 | *floR* | Phenicol resistance | 24392..25606 | ARI-B |
|  | *tetA*(A) | Tetracycline resistance | 26206..27480 |  |
|  | *strB* | Aminoglycoside resistance | 28741..29577 |  |
|  | *strA* | Aminoglycoside resistance | 29577..30379 |  |
|  | *sul2* | Sulphonamide resistance | 30440..31255 |  |
|  | *bla*_CMY-2_ | β-lactam resistance | 60828..61973 | Tn*6538c* |
|  | *aacA7* | Aminoglycoside resistance | 117968..118426 | ARI-A |
|  | *qacED1* | Quaternary ammonium compound resistance | 118556..118903 |  |
|  | *sul1* | Sulphonamide resistance | 118897..119737 |  |
